# Survey, Culture, and Genome Analysis of Ocular *Chlamydia trachomatis* in Tibetan Boarding Primary Schools in Qinghai Province, China

**DOI:** 10.3389/fcimb.2016.00207

**Published:** 2017-01-09

**Authors:** Le Feng, Xinxin Lu, Yonghui Yu, Tao Wang, Shengdong Luo, Zhihui Sun, Qing Duan, Ningli Wang, Lihua Song

**Affiliations:** ^1^State Key Laboratory of Pathogen and Biosecurity, Beijing Institute of Microbiology and EpidemiologyBeijing, China; ^2^Beijing Institute of Ophthalmology, Beijing Tongren Hospital, Capital Medical UniversityBeijing, China

**Keywords:** trachoma, chlamydia, *Chlamydia trachomatis*, *ompA*, China

## Abstract

Trachoma, the leading infectious cause of blindness worldwide, is an ancient human disease. Its existence in China can be traced back to as early as the twenty-seventh century BC. In modern China, the overall prevalence of trachoma has dramatically reduced, but trachoma is still endemic in many areas of the country. Here, we report that 26 (8%) of 322 students from two rural boarding schools of Qinghai province, west China, were identified as having ocular *C. trachomatis* infection; and 15 ocular *C. trachomatis* strains were isolated from these trachoma patients. *Chlamydiae* in 37 clinical samples were genotyped as type B based on *ompA* gene analyses. Three *ompA* variants with one or two in-between SNP differences in the second or fourth variable domain were found. *C. trachomatis* strains QH111L and QH111R were from the same patient's left and right conjunctival swabs, respectively, but their *ompA* genes have a non-synonymous base difference in the second variable domain. Moreover, this SNP only exists in this single sample, suggesting QH111L is a newly emerged *ompA* variant. Interestingly, chromosomal phylogeny analysis found QH111L clusters between a branch of two type B strains and a branch of both A and C strains, but is significantly divergent from both branches. Comparative chromosome analysis found that compared to sequences of reference B/TZ1A828/OT strain, 12 of 22 QH111L's chromosomal genes exhibiting more than nine SNPs have the best homology with reciprocal genes of UGT strains while 9 of 22 genes are closest to those of type C strains. Consistent with findings of UGT-type genetic features in the chromosome, the QH111L plasmid appears to be intermediate between UGT and classical ocular plasmids due to the existence of UGT-type SNPs in the QH111L plasmid. Moreover, the QH111L strain has a unique evolutionarily older cytotoxin region compared to cytotoxin regions of other *C. trachomatis* strains. The genome analyses suggest that the QH111L strain is derived from recombinations between UGT and classical ocular ancestors. This is the first study of culture and characterization of ocular *C. trachomatis* in Qinghai Tibetan areas.

## Introduction

Trachoma is an ancient human disease caused by ocular epithelial infection with serovar A, B, Ba, and C of *Chlamydia trachomatis*. In developing countries and areas of poor hygiene and sanitation, trachoma is the leading reason of preventable blindness (Whitcher et al., 2001). The world health organization aims to eliminate blinding trachoma by 2020 through SAFE strategy—Surgery for trichiasis, Antibiotics for infection, Facial cleanliness, and Environmental improvements (Cook, 2008). Mass administration of azithromycin has been applied in 28 countries in Africa and Asia (Kurylo, 2013). Much progress has been made toward trachoma elimination in some countries (Lavett et al., 2013). However, sustainable accomplishment of the 2020 global goal is doubtful due to lacking more sustainable measures such as a trachoma vaccine and the incomplete coverage of SAFE implementation in unidentified trachoma endemic communities.

The record of trachoma history in China dates back to 2679 B.C. The heavy prevalence of trachoma in China rural areas was vividly reflected by a Chinese saying “Nine out of ten people having sand in eye.” Sand eye in Chinese literally means trachoma. The trachoma pandemic continued until the late 1970s when China started its economic reform. With economic growth and implementation of plans for trachoma control by the central government (Wang N. et al., 2016), the overall prevalence of trachoma in China was dramatically reduced. In May 2016, the Chinese government announced the elimination of blinding trachoma after a trachoma survey covering 16 provinces where previously trachoma prevalence was highest (Wang N. et al., 2016). Based on this survey, the prevalence of active trachoma in 1–9 year olds and trichiasis in adults over 14 years are 0.196 and 0.002%, respectively.

The announcement of blinding trachoma elimination by the Chinese government defines that trachoma is not a significant public health problem in China. However, active trachoma still remains in many focal areas of China including Gansu, Hunan, Hubei, Liaoning, Ningxia, Hebei, Inner Mongolia (Zhou et al., 2007; Wang, 2012; Dong and Liu, 2013; Yu et al., 2014; Zhao, 2015), and further research on trachoma in mainland China is urgently needed in order to understand the evolution and epidemiology of trachoma and ocular *C. trachomatis* in China (Wang, 2016). Since the first isolation and culture of *C. trachomatis* using chicken embryonic eggs by Tang et al. (1956), 46 *C. trachomatis* strains were isolated from patients of Beijing, Guangdong and Jinan before 1970 (Zhang, 1977). Unfortunately, these early strains were lost and their DNA not available for genetic analysis. In 1991 and 2007, *C. trachomatis* genotypes B and C were found in primary school students from north China and the ratio of genovars B and C infections was about 4 to 1 (Zhang et al., 1991; Zhou et al., 2007). China holds a vast territory, and in most areas ocular *C. trachomatis* requires further investigation.

Prevalence of trachoma and ocular *C. trachomatis* in west China is currently under studied. Suspected active trachoma cases were found during a visit to boarding schools in Galeng town, Xunhua county, Qinghai province in 2014 by trachoma rapid assessment. Two separate visits to two Tibetan boarding schools in Galeng town were made in 2015 for trachoma screening and sample collection. Here, we report further molecular analysis and the isolation of ocular *C. trachomatis* in these two rural Tibetan boarding schools. We report the first genome sequence of *C. trachomatis* isolated from a conjunctival sample of an 11 year old boy with trachoma. This study has important implications for trachoma control in China and for understanding evolution and epidemiology of trachoma.

## Materials and methods

### Ethics statement

This study was approved by the Capital Medical University ethics committee in accordance with the medical research regulations of China. Consent was obtained from the Qinghai Province People's Government prior to entry into the schools. All participants were aged 6–13 years. Consent for examination and to have specimens collected was given on their behalf by a legally responsible guardian. All samples were anonymous.

### Clinical examination and sample collection

Clinical examination and grading of trachoma was made by ophthalmologists and is reported using the equivalent scales of the WHO trachoma simplified grading system. Swab samples of conjunctivas (left and right eye) or conjunctival sacs from trachoma patients and non-trachoma students were collected as previously described (Li et al., 2016). All swabs were stored in ice boxes in the field and were transported to laboratories by air within 48 h of collection. Samples for chlamydial isolation and culture were mixed with equal volumes of 1X Sucrose Phosphate Glucose (SPG) buffer and were then stored at −80°C.

### Isolation and culture of *C. trachomatis*

Buffalo Green Monkey Kidney (BGMK) cell monolayers in 96-well-plates were infected with 100 μl (1/20 of total volume) of samples per well by centrifugation at 800 g for 30 min. Cells were then incubated in DMEM-10 (containing 1 μg/ml cycloheximide, 100 μg/ml vancomycin, 5 μg/ml amphotericin B, and 10 μg/ml gentamycin) for 72 h at 37°C in 5% CO_2_ (Caldwell et al., 1975). Cultures were successively passaged three times and were observed using phase microscopy every 48 h post-infection.

### Genome sequencing

*C. trachomatis* strain QH111L was grown on BGMK cells. Genomic DNA was isolated from partially purified EBs, as previously described (Carlson et al., 2005). The DNA was sequenced on an Illumina HiSeq 2500 platform through PE250 protocol, providing about 630X coverage. *De novo* assembly of sequencing reads were performed using Edena (Hernandez et al., 2008). ORFs were identified and annotated with Prodigal (Hyatt et al., 2010).

### Genome wide SNP analysis

All reads were mapped to reference sequence using Burrows-Wheeler Aligner (Li and Durbin, 2009). The alignment results were analyzed using SAMTools (Li et al., 2009). SNPs were called only for variable positions with a minimum mapping quality of 30. The minimum and maximum read depths were set at 20 and 1000, respectively.

### *ompA* and genome phylogeny analysis

Maximum likelihood was used to reconstruct the phylogeny of a Qinghai isolate from this study and reference isolates. Sequences were mapped to *C. trachomatis* A/Har13 using SAMtools (Li et al., 2009). SNPs were called as described previously (Harris et al., 2012). Phylogenies were computed with RAxML (Stamatakis et al., 2008) from a variable sites alignment using a GTR+gamma model and are midpoint rooted.

### Phylogenetic analysis of the cytotoxin region

The evolutionary history was inferred by using the Maximum Likelihood method based on the Tamura-Nei model (Tamura and Nei, 1993). The tree with the highest log likelihood (−18992.4199) is shown. Initial tree(s) for the heuristic search were obtained automatically by applying Neighbor-Join and BioNJ algorithms to a matrix of pairwise distances estimated using the Maximum Composite Likelihood (MCL) approach, and then selecting the topology with superior log likelihood value. The tree is drawn to scale, with branch lengths measured in the number of substitutions per site. The analysis involved six nucleotide sequences. All positions containing gaps and missing data were eliminated. There were a total of 8061 positions in the final dataset. Evolutionary analyses were conducted in MEGA7 (Kumar et al., 2016).

### Nucleotide sequence accession numbers

The genome sequence of B/QH111L has been deposited in the GenBank database under accession numbers CP018052 (chromosome) and CP018053 (plasmid). The original sequencing data has been deposited to the Sequence Read Archive under bioproject PRJNA352770.

## Results

### Clinical examination and sample collection

Tibetan students aged 6–13 years old in two boarding primary schools (Jianshetang primary school and Galeng central primary school) in Galeng town, Xunhua county, Qinghai province, were assessed for trachoma using WHO trachoma simplified grading system. Both eyes of 322 students were examined for the clinical signs of active trachoma. There were no cases of papillary hypertrophy that were equivalent to Trachomatous inflammation-intense (TI) according to the WHO simplified grading system. Twenty-six of 322 (8%) examined had five or more follicles [Trachomatous Inflammation-follicular (TF)] in one or both eyes. A further 19 individuals had between one and four follicles on either or both everted upper conjunctival surfaces. In total 45/322 (14%) were considered to have evidence of conjunctival follicles that may be indicative of active trachoma. A total of 115 trachoma swabs from each conjunctiva with one or more follicles and the corresponding conjunctival sac were collected. All samples were examined using a commercial qPCR kit detecting chlamydial plasmid and *ompA* sequencing for qPCR-positive conjunctiva swabs was performed, as previously described (Li et al., 2016; Wang N. et al., 2016). Forty-five swabs from 26 students were qPCR-positive for *C. trachomatis* infection and two type-B *ompA* variants were identified.

### Isolation and culture of ocular *C. trachomatis*

No previous studies have been performed on ocular *C. trachomatis* in west China and in Tibetan people. Many regions in west China are less developed and the population may remain at increased risk of trachoma. In addition, studies of ocular *C. trachomatis* have been neglected in China since the 1980s (Cheng et al., 2011). We therefore sought to first rapidly assess if trachoma and *C. trachomatis* ocular infection was present in the community and if possible isolate and culture *C. trachomatis* for future pathogen-related studies. By using BGMK cells, 15 *C. trachomatis* cultures were generated from seven trachoma patients (all of which were positive by *C. trachomatis* qPCR). Numbers of these strains were assigned as follows: QH111R, QH111L, QH113R, QH118L, QH118S, QH120S, QH123R, QH123S1, QH123S2, QH155R, QH155L, QH155S, QH159R, QH159L, and QH159S (QH, QingHai; 111–159, patient ID; L or R, Left or Right Conjunctiva; S, Conjunctival Sac). Detailed backgrounds of these 15 strains are summarized in Table 1.

**Table 1:** **Backgrounds of 15 ***C. trachomatis*** strains from Qinghai, China**.

**Patient ID**	**Gender (F/M)**	**Age (Y)**	**Follicle counts**		**Chlamydia strains**		
			**OD**	**OS**	**OD**	**OS**	**CS**
111	M	11	>5	>5	QH111R	QH111L	ND
113	M	10	5	5	QH113R	–	ND
118	F	7	3	4	–	QH118L	QH118S
120	M	8	>5	5	–	–	QH120S
123	M	7	5	5	QH123R	–	QH123S1
155	F	11	2	3	QH155R	QH155L	QH155S
159	M	12	5	5	QH159R	QH159L	QH159S

*OD, right eye; OS, left eye; CS, conjunctiva sac; –, culture negative; ND, no data*.

### Identification of three *ompA* variants from a same type-B ancestor with a UGT-type codon

**Figure 1:**
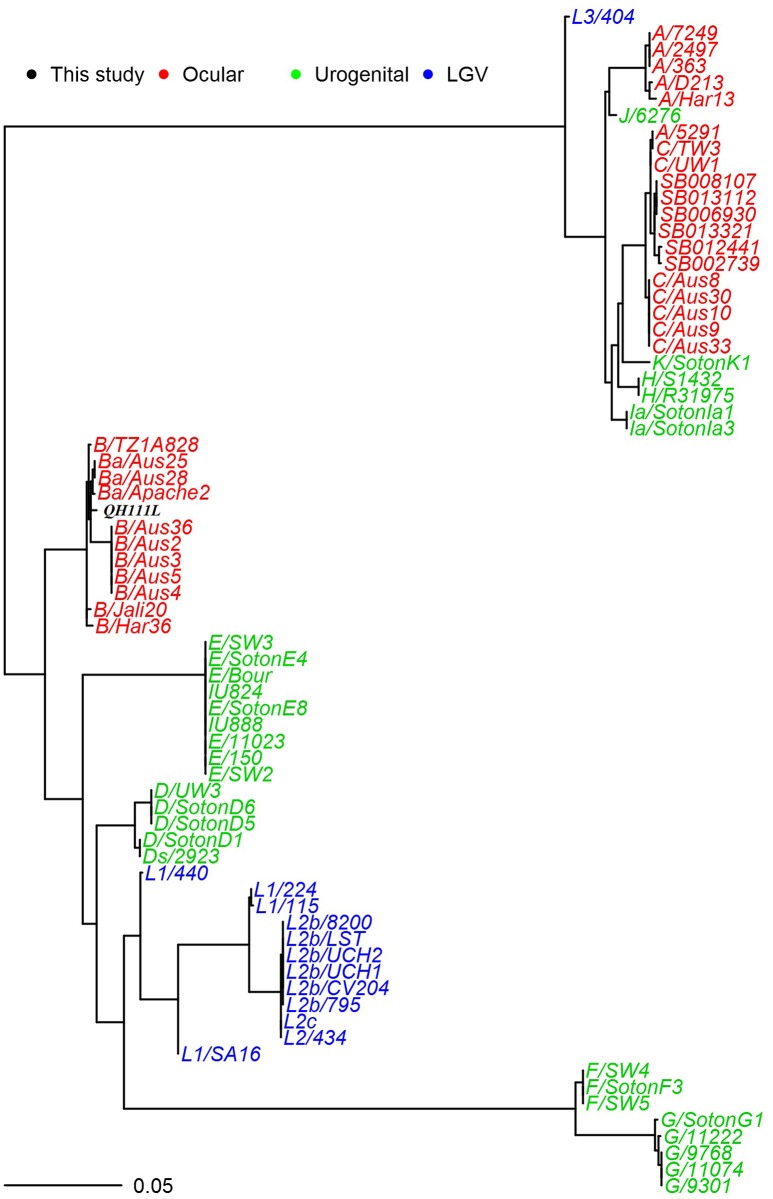
**Maximum likelihood reconstruction of the ***ompA*** phylogeny of sequences from QH111L and reference isolates**. Sequences were mapped to *C. trachomatis* A/Har13 using SAMtools (Li et al., 2009). SNPs were called as described (Harris et al., 2012). Phylogenies were computed with RAxML (Stamatakis et al., 2008) from a variable sites alignment using a GTR+gamma model and are midpoint rooted. The scale bar indicates evolutionary distance. The sequences collected in this study are colored black, reference isolates are colored by tissue tropism (red, ocular reference sequences; green, urogenital reference sequences; blue, LGV reference sequences).

Li et al. previously reported the identification of two type-B *ompA* variants in 37 qPCR-positive conjunctival samples (Li et al., 2016). Here, we report the finding of another *ompA* variant in a single sample of no. 111 patient's left conjunctiva, which has a non-synonymous SNP in *ompA* VD-II compared to *ompA* of the same patient's right conjunctiva. This finding was confirmed by *ompA* sequencing of *C. trachomatis* strains QH111L and QH111R. Here we arbitrarily assigned different *ompA*s of QH111L, QH111R and QH118L as representatives of the variants. Compared to *ompA* of QH111R, *ompA*s of QH111L and QH118L have one non-synonymous SNP at 511 in VD-II and at 887 in VD-IV, respectively (Figure S1). The minor changes in *ompA*s indicate the Qinghai strains share a common ancestor. Phylogenetic analysis of *ompA*s from the QH111L and reference strains found that the Qinghai strain clusters tightly with previous B and Ba strains, consistent with the known high conservation of type-B *ompA*s (Figure 1).

**Figure 2:**
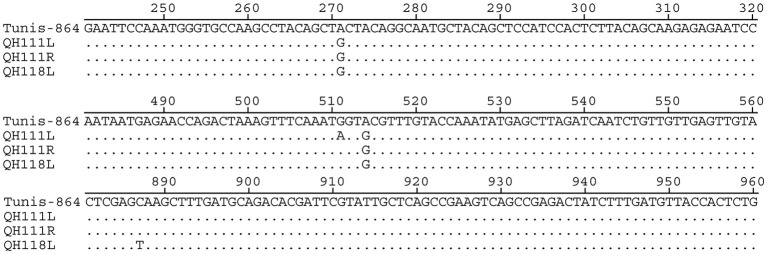
**Alignment of ***ompA***s of the B/Tunis-864, QH111L, QH111R, and QH118L strains**.

BLAST analyses revealed that *ompA*s of the Qinghai strains have the highest homology to *ompA* of B/Tunis-864. Compared to *ompA* of B/Tunis-864, *ompA*s of QH111L, QH111R, and QH118L have 3, 2, and 3 SNPs in *ompA* VD-I, -II, or -IV, respectively (Figure 2). Interestingly, the codon GCT at alanine 91 which contains one of two shared SNPs is previously only found in *ompA*s of UGT-type *C. trachomatis* strains including LGV, F, and G types (Figure S2). Given the high conservation of type-B *ompA*s and the below findings of UGT-type genetic features in both the plasmid and chromosome of QH111L, this UGT-type codon is an interesting marker of the Qinghai trachoma strains. Moreover, based on *ompA* sequencing, 37 conjunctival samples can be classified into three arbitrarily named groups -QH111L, QH111R, and QH118L, containing 1, 30, and 6 samples, respectively. The different group members and the different numbers of SNPs compared to B/Tunis-864 suggest that QH111R is the dominant strain while QH118L is an established variant and QH111L is a newly emerged variant.

### Phylogeny and comparison analysis of *C. trachomatis* QH111L chromosome

**Figure 3:**
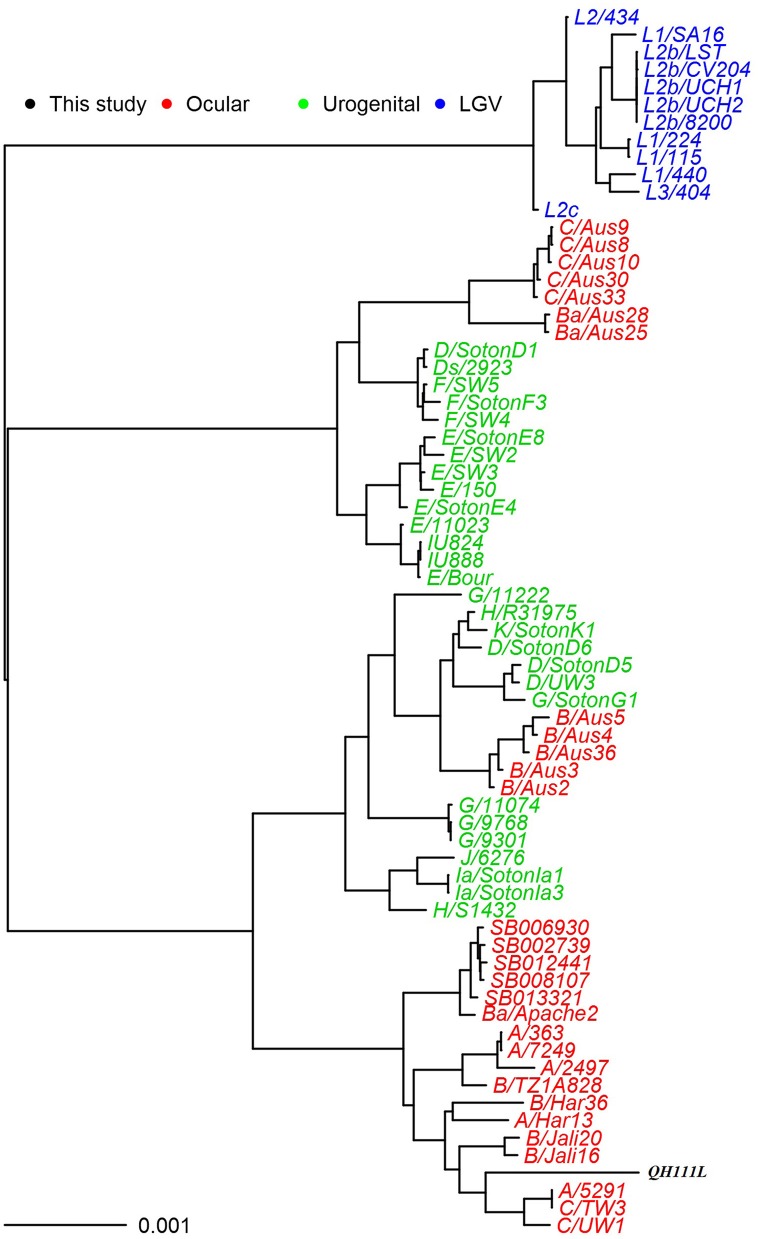
**Maximum likelihood reconstruction of the whole-chromosome phylogeny of the QH111L and reference isolates**. Sequences were mapped to *C. trachomatis* A/Har13 using SAMtools (Li et al., 2009). SNPs were called as described (Harris et al., 2012). Phylogenies were computed with RAxML (Stamatakis et al., 2008) from a variable sites alignment using a GTR+gamma model and are midpoint rooted. The scale bar indicates evolutionary distance. The sequences collected in this study are colored black, reference isolates are colored by tissue tropism (red, ocular reference sequences; green, urogenital reference sequences; blue, LGV reference sequences).

To understand Qinghai strains' evolutionary relationships among *C. trachomatis* strains with available genomic data, we sequenced QH111L strain's whole genome and constructed a whole chromosomal phylogeny using genome-wide SNPs (Figure 3). The resulting tree shows that QH111L falls in the classical ocular lineage, clusters between a branch of two B strains and a branch of both A and C strains, and was significantly divergent from both branches. This contrasts with classification of Qinghai strains as type B by *ompA* genotyping. The usefulness of *ompA* for evolutionary analysis of *C. trachomatis* is unreliable when recombinations occur in *ompA* and other regions of the *C. trachomatis* genome (Harris et al., 2012). In the genome phylogeny, previous type A and B strains fall in multiple branches, suggesting different recombination events between these two type strains. The tight clustering of A/5291 and C/TW3 is indicative of recombinations between A/C type strains. Recombinations between the B and C type strains are suggested by the intermediate position of QH111L between the type C branch and branches of other B strains.

To understand the B/QH111L strain's significant divergence from other type B strains, SNP comparisons between QH111L and the evolutionary old reference B/TZ1A828 were performed. A total of 2128 SNPs were identified between these two strains' chromosomes. One thousand eight hundred and eighty-six SNPs localized within coding (ORF) regions. A total of 22 ORFs exhibits more than nine SNPs (Table 2). BLAST analysis of these ORFs of QH111L found that nine genes including 197 SNPs are closest to reciprocal genes of type C strains, consistent with QH111L's close clustering to type C strains. Eight of these C type genes fall in two clusters—CT144–147 and CT677–680. Intriguingly, 12 of these 22 ORFs, including a CT049–51 cluster, of QH111L including 405 SNPs have the highest homology with those of UGT strains. Phylogenetic analyses of the CT049–051 cluster of the QH111L strain find that the QH111l genes constantly cluster with those of previous UGT strains (Figures S3–S5). Thus, genome comparison analyses further revealed UGT-type and C-type genes in QH111L, which likely explains the significant divergence of the QH111L strain.

**Table 2:** **Best BLAST hits of all QH111L genes harboring ≥10 SNPs compared to those of B/TZ1A828/OT**.

**ORF**	**Function**	**SNP counts**	**Best BLAST hits**
CT049	Hypothetical protein	54	D/G/Ia
CT050	Hypothetical protein	94	D/G/Ia
CT051	Hypothetical protein	76	D/K
CT082	Hypothetical protein	13	C/B
CT144	Hypothetical protein	11	C/A
CT145	*pkn1*	11	C
CT146	*dnlJ*	10	C
CT147	Hypothetical protein	48	C
CT157	Phospholipase D endonuclease superfamily	19	G
CT166	Hypothetical protein	12	D/E/F/K
CT214	Hypothetical protein	17	D/E/F/G
CT394	*hrcA*	16	G/Ia/J
CT414	*pmpC*	13	G
CT456	*tarp*	11	B
CT545	*dnaE*	10	G
CT677	*rrf*	15	C/A
CT678	*pyrH*	23	C
CT679	*tsf*	56	C/A
CT680	*rpsB*	10	C
CT682	*pbpB*	60	G
CT684	*sufB*	13	Ia/J
CT837	Hypothetical protein	10	Ia/J

### Phylogeny and comparison analysis of *C. trachomatis* QH111L plasmid

**Figure 4:**
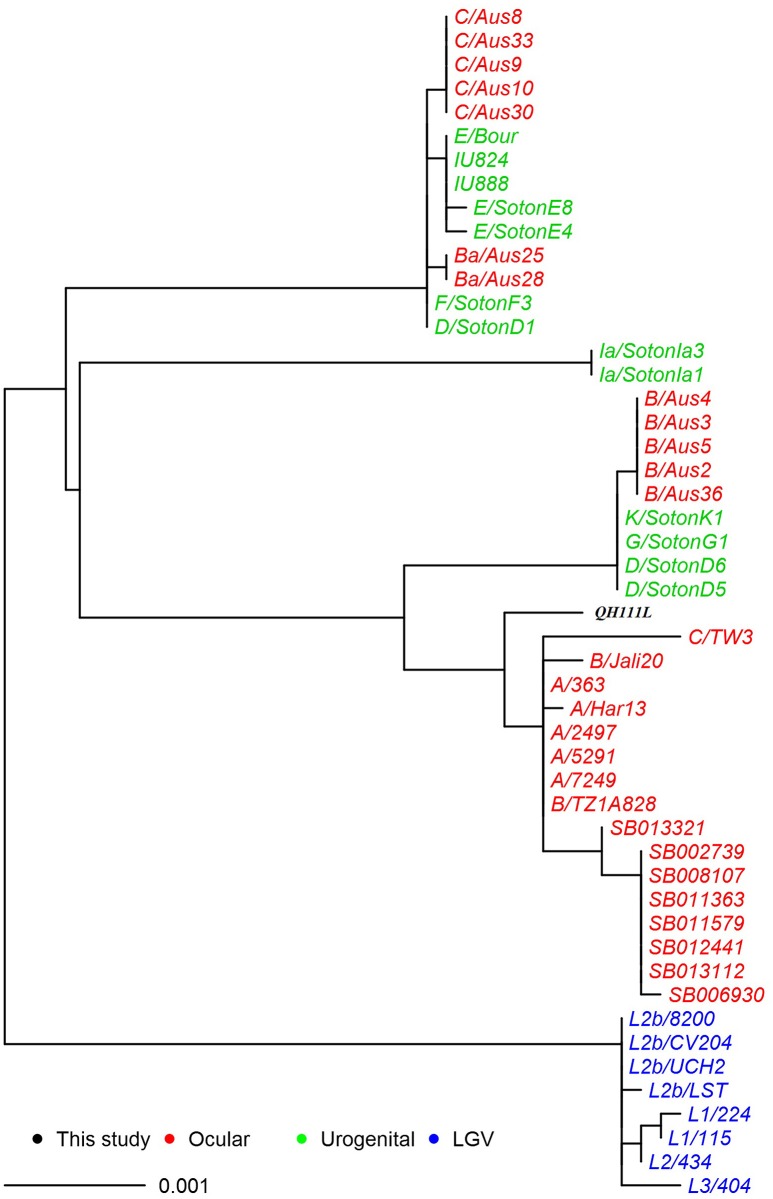
**Maximum likelihood reconstruction of the plasmid phylogeny of the QH111L and reference isolates**. Sequences were mapped to *C. trachomatis* A/Har13 using SAMtools (Li et al., 2009). SNPs were called as described (Harris et al., 2012). Phylogenies were computed with RAxML (Stamatakis et al., 2008) from a variable sites alignment using a GTR+gamma model and are midpoint rooted. The scale bar indicates evolutionary distance. The sequences collected in this study are colored black, reference isolates are colored by tissue tropism (red, ocular reference sequences; green, urogenital reference sequences; blue, LGV reference sequences).

The co-evolution of chlamydial plasmid and chromosome is well-established in *C. trachomatis* except for the Ba/Apache2 strain (Seth-Smith et al., 2009; Harris et al., 2012). A similar phylogeny based on plasmid SNPs was constructed in order to understand Qinghai strain's evolutionary history (Figure 4). Notably, the QH111L plasmid belongs to the classical ocular lineage, is closest to plasmids of type C strains but appears to be intermediate between UGT and classical ocular plasmids. The plasmid sequence of *C. trachomatis* is highly conserved. Compared to the plasmid of B/TZ1A828, the QH111L plasmid only has six SNPs (Table 3). However, two of these SNPs in *pgp2* and *pgp5*, respectively, can only be found in plasmids of previously reported UGT strains. Thus, the phylogenetic position of QH111L plasmid is congruent with the phylogeny of whole chromosome in the aspect of finding an ocular-type genetic backbone and UGT-type characteristics in the QH111L strain; the co-evolution of plasmid and chromosome in the QH111L strain is correspondingly supported.

**Table 3:** **SNPs in the QH111L plasmid compared to plasmid of the B/TZ1A828 strain (NC_012627)**.

**Gene ID**	**Nucleotide position**	**Nucleotide change**	**Amino acid change**	**Function**
*pgp1*	3463	T > G	Ser > Ala	*dnaB*
	3464	C > T	Ser > Leu	
*pgp2*	3798	A > G	Ile > Val	Hypothetical protein
*pgp3*	4840	A > G	Thr > Ala	Secreted effector
*pgp4*	5581	G > T	Val > Leu	Transcriptional regulator
*pgp5*	6335	T > C	Val > Ala	*parA* family

Another interesting finding was a unique non-synonymous SNP (19T) in *pgp4* (Table 3). Pgp4 is a master transcriptional regulator of both plasmid and chromosomal virulence-associated genes and thus an important virulence factor (Song et al., 2013). The *pgp4* gene is highly conserved as currently known *C. trachomatis pgp4* genes only have one SNP at base 15 between genes of non-LGV (15A) including QH111L and LGV (15C) strains (Figure S6). The unique SNP in *pgp4* of QH111L possibly correlates with the finding of high polymorphisms in plasmid regulated genes—CT049 (UGT-type) and CT144 (C-type).

### Tropism-associated genetic features in the QH111L strain

Several putative tropism loci relating with respect to ocular or UGT infections have been previously described, such as *ompA* (Andersson et al., 2016), the *trp* operon (Caldwell et al., 2003), *tarp* (Lutter et al., 2010), *pmp* (Carlson et al., 2005; Andersson et al., 2016), cytotoxin (Carlson et al., 2004), and the plasmid (Seth-Smith et al., 2009). The sequence of QH111L suggests that this isolate is a classical ocular strain with UGT-type features in *pmpC*, the plasmid, and the plasmid-regulated CT049 gene. Interestingly, the *trp* operon of QH111L has the best homology to those of UGT strains (Figure S7), but similar to the *trp* malfunction of ocular strains, has disrupted *trpB* and *trpA*. However, different from previous classical ocular strains (Caldwell et al., 2003), the disruptions of *trpB* and *trpA* in QH111L are due to an internal 20 bp deletion in *trpB*, and an early stop codon and an internal 112 bp deletion in *trpA*.

Another potential genetic feature related with tropism in QH111L is the truncation of CT442 or *crpA*, a 15-kDa cysteine rich outer membrane protein of unknown function (Bannantine et al., 2000). The CT442 of QH111L truncates after amino acid 19 due to a non-synonymous mutation (C to T). Similar truncations of this gene are described in C/TW-3 and C/UW-1 strains, due to deletions and insertions, respectively (Borges et al., 2014). So far, the truncation of CT442 is only described in ocular strains, this might suggest a relationship with infection tropism or preference to ocular mucosa.

**Figure 5:**
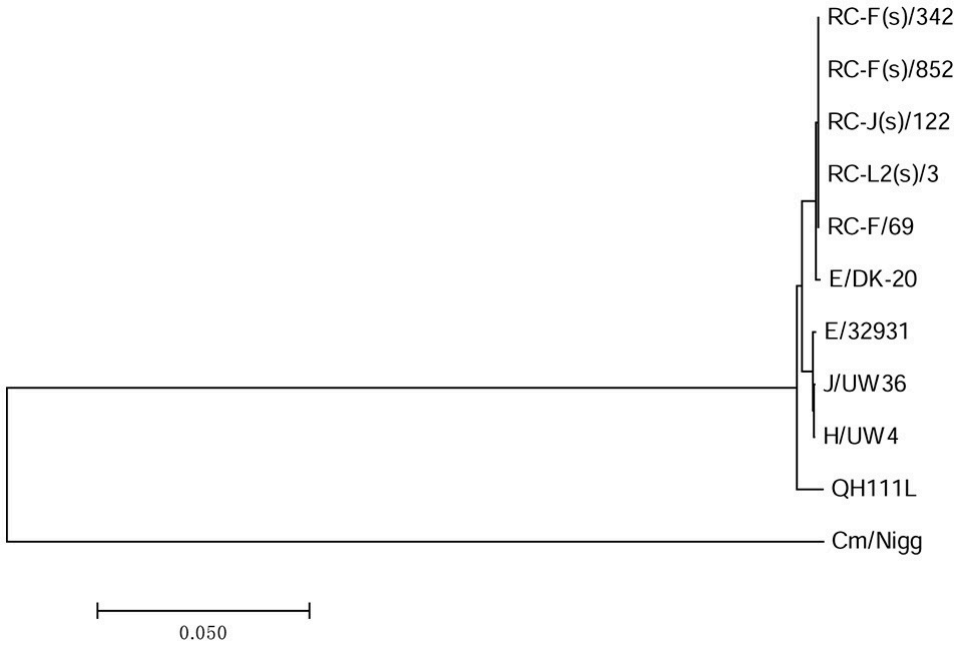
**Maximum likelihood reconstruction of the cytotoxin-region phylogeny of the QH111L and reference isolates**.

One important locus associated with tropism of ocular and UGT infections is the cytotoxin (Carlson et al., 2004). Compared to cytotoxin genes of *C. muridarum*, the cytotoxin genes of *C. trachomatis* are extensively interrupted by mutations and deletions. Surprisingly, in contrast to previous ocular strains, the QH111L genome contains a large cytotoxin region albeit with interruptions. The length of the cytotoxin region of QH111L is 9440 bp, containing the ~4970 bp region deleted in previous ocular strains (Carlson et al., 2004). Phylogenetic analysis of this region suggests that the cytotoxin region of QH111L is evolutionarily older than those of other *C. trachomatis* UGT strains (Figure 5). This finding of a large cytotoxin region highlights the unique genetic features of the QH111L strain.

## Discussion

This work presents the first culture and genome analysis of ocular *C. trachomatis* in Tibetan children from Qinghai province, west China. It could have important implications for trachoma control in provincial regions of China and for understanding evolution and epidemiology of ocular *C. trachomatis*.

The first and the most widely sampled trachoma survey in China took place between 2012 and 2014 and concluded that China had reached the elimination threshold targets (Wang N. et al., 2016). Children resident in boarding schools investigated in this study were found to have clinical evidence of active trachoma (conjunctival lymphoid follicles) during this survey. Thus, a complete trachoma screening and sampling was performed in order to understand the prevalence and etiology of trachoma in these schools (Li et al., 2016; Wang M. et al., 2016). The Wang group identified 45 cases of follicular conjunctivitis, 26 of them were infected with *ompA* genovar B *C. trachomatis*, and identified other potential follicle-causing bacteria in both *C. trachomatis*-positive and—negative cases (Li et al., 2016; Wang, 2016; Wang M. et al., 2016; Wang N. et al., 2016). Their identification of non-chlamydial bacteria in clinically identified cases of follicular trachoma is congruent with other studies (Burton et al., 2011; Burr et al., 2013).

The overall decrease in trachoma prevalence in China is an event accompanied by the country's economic development. Though trachoma is not regarded as an important health burden, trachoma reports are reasonably frequent in Chinese biomedical literature. In recent trachoma surveys, trachoma has been reported in primary and middle school students from many areas of China (Zhou et al., 2007; Wang, 2012; Dong and Liu, 2013; Yu et al., 2014; Zhao, 2015). All these reports have no description of intense inflammatory trachoma or more severe trachoma cases, likely due to the low prevalence of trachoma in these areas. Given that presence of clinical signs of trachoma such as conjunctival follicles might be due to non-chlamydial causes in low prevalence settings or regions (Butcher et al., 2016), the use of a specific nucleic acid amplification test or preferably whole-genome sequencing is recommended in order to establish or rule out the cause of disease.

One implication from this and previous work (Boost and Cho, 2005; Dong and Liu, 2013; Yu et al., 2014; Xue et al., 2016) is that boarding schools in rural areas and migrant schools in cities could be foci of disease and infection and could be a barometer for disease in the communities from which they originate. This information could then be used to direct structured or population based trachoma surveys in more remote or challenging regions or communities in China. Two separate studies reported higher prevalence of trachoma in boarding schools (Boost and Cho, 2005; Yu et al., 2014). A recent study found trachoma to be prevalent in migrant schools of Shanghai, unfortunately without performing *C. trachomatis* PCR or genotyping (Xue et al., 2016). The fast growth of boarding and migrant schools in China is a feature of the country's developing society in rural areas and cities, respectively, as young parents work in urban industries and depend on boarding or migrant schools for their children's education. The crowded living facilities and poor hygiene practices possibly contributing to continued transmission of infection and continued observation of the clinical signs of trachoma within these schools.

Previous work of culturing and typing ocular *C. trachomatis* was conducted in Han people of east China. Both B and C type *ompA* genovar strains have been consistently found in mainland China (Zhang et al., 1991; Zhou et al., 2007). From our small sample of specimens we found *ompA* B genovars in Tibetans. Whether Tibetan B genovars have a close evolutionary relationship with the B strains from east mainland China is unknown due to lacking genomic data from the latter. The *ompA* of Tibetan strains have the highest homology to that of the geographically distant B/Tunis strain, not the neighboring Taiwan strain B/TW5. Our single whole-genome sequence currently available from this study clusters with the Taiwan strain C/TW3 by whole genome phylogeny.

Our understanding of the origin and evolution of ocular *C. trachomatis* depends on genome analyses. Current known strains of ocular tropism include strains from the classical ocular lineage and Australian trachoma strains falling in the UGT lineages (Harris et al., 2012; Andersson et al., 2016). The classical ocular lineage was hypothesized to have evolved only once. The Australian trachoma strains emerged from frequent recombinations between the classical ocular strains and UGT strains at *ompA* and *pmpEFGH*, retained chromosome and plasmid backbones of UGT strains and the capability of spreading through UGTs (Andersson et al., 2016). Similar to Australian trachoma strains, the Qinghai strain QH111L appears to be derived from recombinations between B and C strains of the classical ocular lineage and UGT strains, as the QH111L strain falls between the B and C sublineages in genome phylogeny but has UGT-type features in *ompA*, plasmid, plasmid-regulated chromosomal genes, and other chromosomal genes.

The antiquity of the Qinghai QH111L strain is also implied in the plasticity zone of its chromosome—containing disrupted *trpB* and *trpA* and especially relics of the large cytotoxin. The finding of near full-length cytotoxin sequences in QH111L adds the diversity of the plasticity zone of ocular strains. To be noted, the cytotoxin fragment -CT166 in QH111L is disrupted as in the C/TW3 and A/5291 strains. Whether other cytotoxin fragments in QH111L still function is unknown. Previously, the long-length version of cytotoxin was only found in a limited of non-ocular *C. trachomatis* strains (Carlson et al., 2004; Putman et al., 2013). Importantly, the cytotoxin locus of QH111L is phylogenetically older than those of other non-ocular strains. If the decay of this locus is a unidirectional deletion event, the cytotoxin region of the Qinghai strain is a relic of early ancestors of both ocular and UGT *C. trachomatis* strains.

This work is an example of surveying trachoma in a focused setting in provincial China. Given the county's vast territory, *C. trachomatis* genomic data from other areas of mainland China are required. This will likely assist our understanding of ocular *C. trachomatis* evolution, during the window of complete elimination of trachoma both in China and in a global context.

## Author contributions

LF, YY, TW, SL, and ZS: data acquisition, data analysis, data interpretation, and revising of the manuscript; XL, QD, and NW: clinical sample acquisition, data acquisition, data analysis, data interpretation, and revising of the manuscript; LS: data acquisition, data analysis, data interpretation, writing of the manuscript, revising of the manuscript, and principle investigator.

## Funding

This work was supported by the National Natural Science Foundation of China (No. 31570177), the Foundation of State Key Laboratory of Pathogen and Biosecurity (No. SKLPBS1409), and the National Key Research Project of China (No. 2016YFC1202500 and 2016YFC1202705).

### Conflict of interest statement

The authors declare that the research was conducted in the absence of any commercial or financial relationships that could be construed as a potential conflict of interest.

## Supplementary material

Figure S1 **Alignment of three ***ompA*** variants of ocular ***C. trachomatis*** from Qinghai, China**.

Figure S2 **Partial alignment of available ***ompAs*** from GenBank**.

Figure S3 **A fast-minimum-evolution tree produced by BLAST analysis of the QH111L CT049 gene**.

Figure S4 **A fast-minimum-evolution tree produced by BLAST analysis of the QH111L CT050 gene**.

Figure S5 **A fast-minimum-evolution tree produced by BLAST analysis of the QH111L CT051 gene**.

Figure S6 **Partial alignment of available ***pgp4*** sequences from GenBank**.

Figure S7 **A fast-minimum-evolution phylogenetic tree produced by BLAST analysis of the QH111L ***trp*** operon**.
