# Supplementary figures and images for: Survey, Culture, and Genome Analysis of Ocular Chlamydia trachomatis in Tibetan Boarding Primary Schools in Qinghai Province, China
Source: Front Cell Infect Microbiol. 2017 Jan 9;6:207. doi: 10.3389/fcimb.2016.00207 (PMC5220689; doi:10.3389/fcimb.2016.00207)

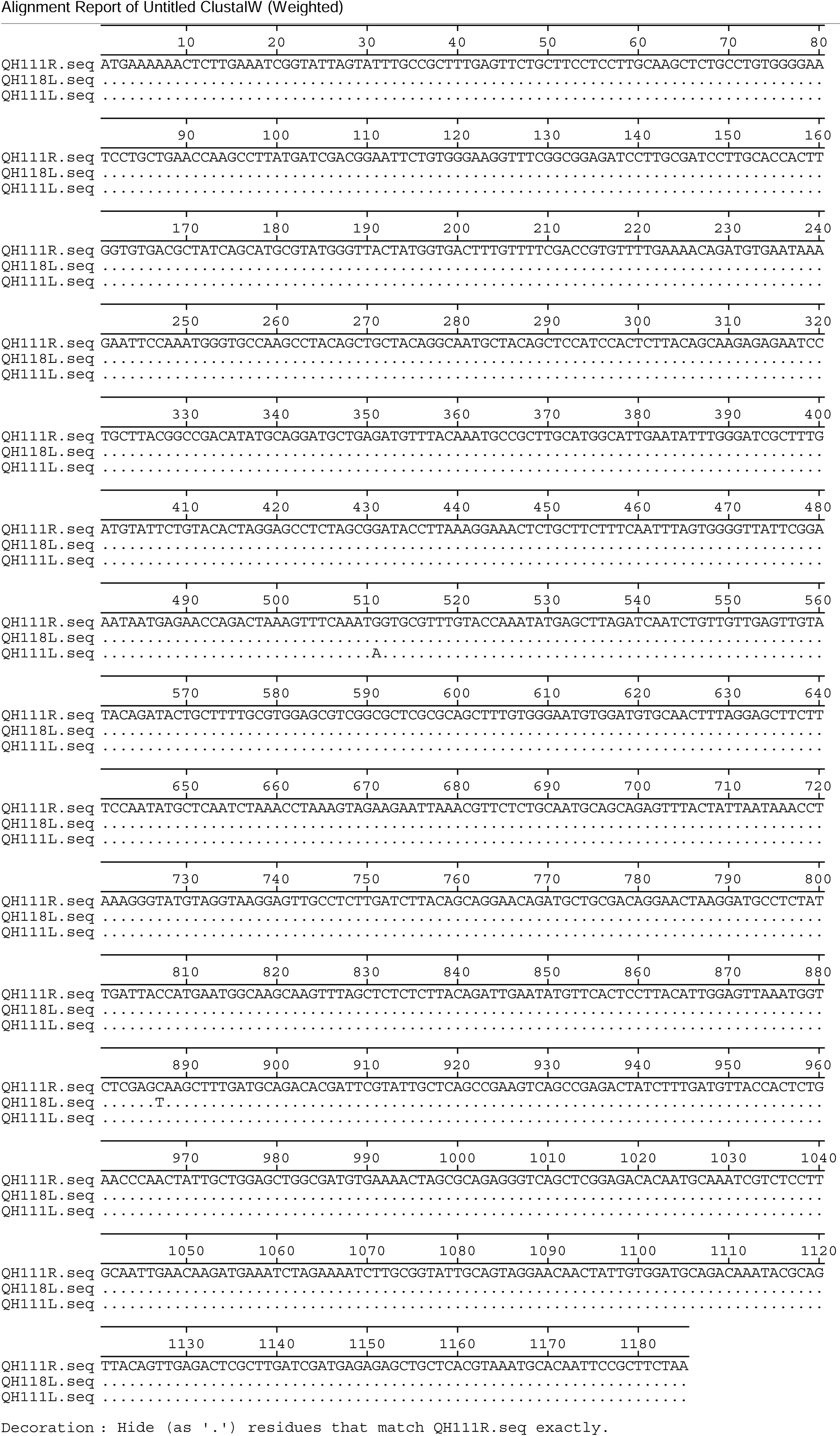

Supplement: Figure S1 — Alignment of three ompA variants of ocular C. trachomatis from Qinghai, China. [file Image1.TIF]

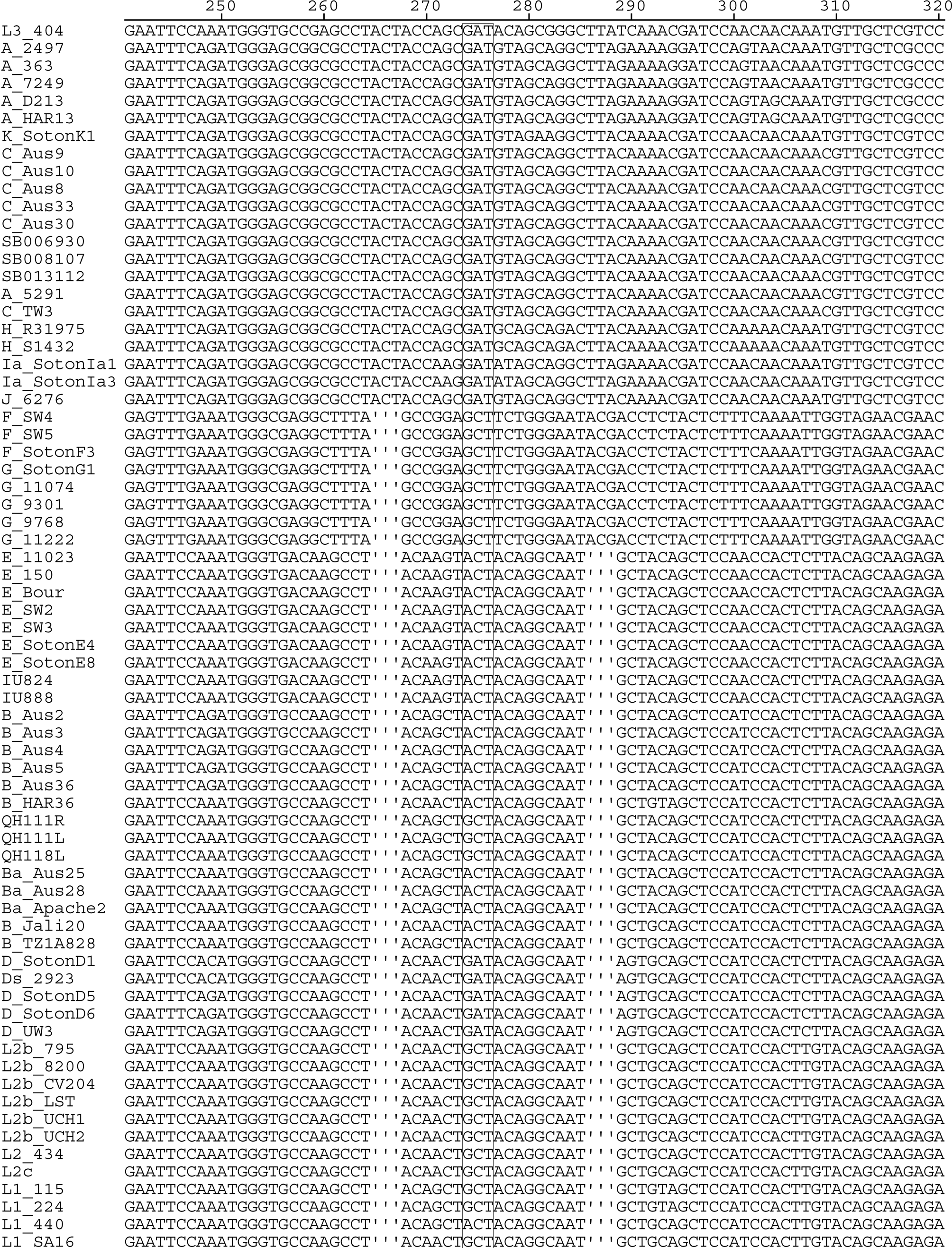

Supplement: Figure S2 — Partial alignment of available ompAs from GenBank. [file Image2.TIF]

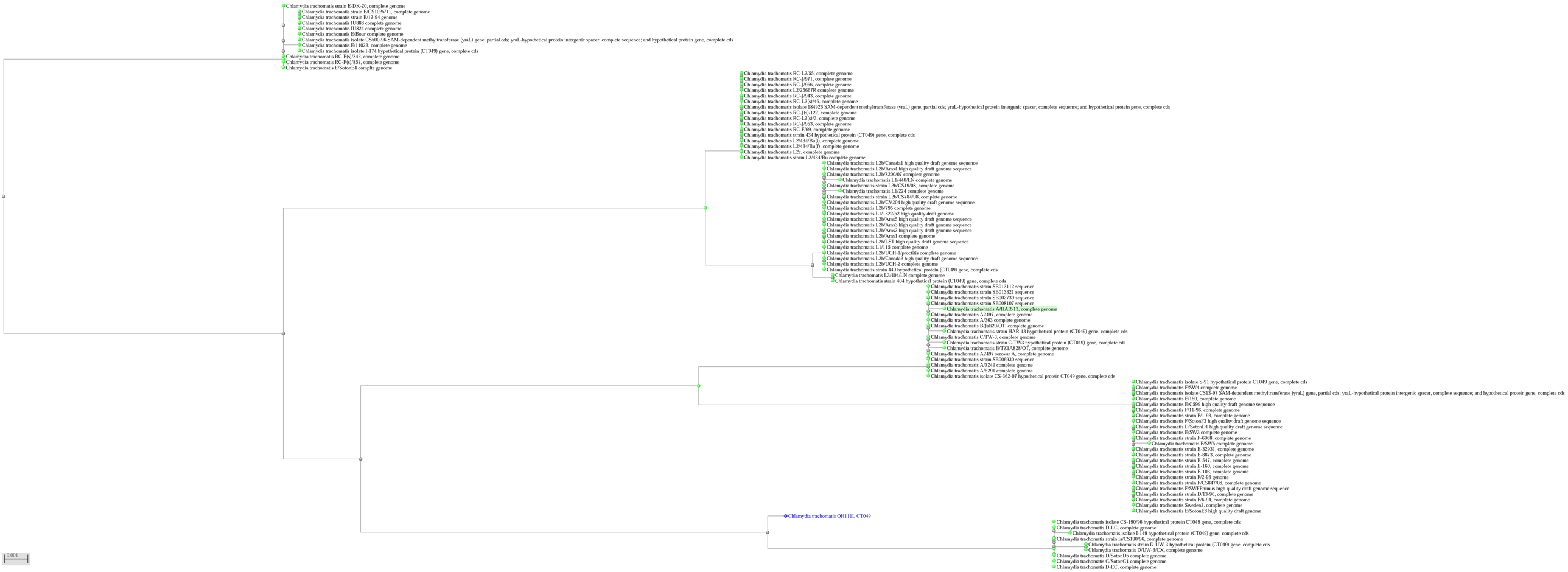

Supplement: Figure S3 — A fast-minimum-evolution tree produced by BLAST analysis of the QH111L CT049 gene. [file Image3.TIF]

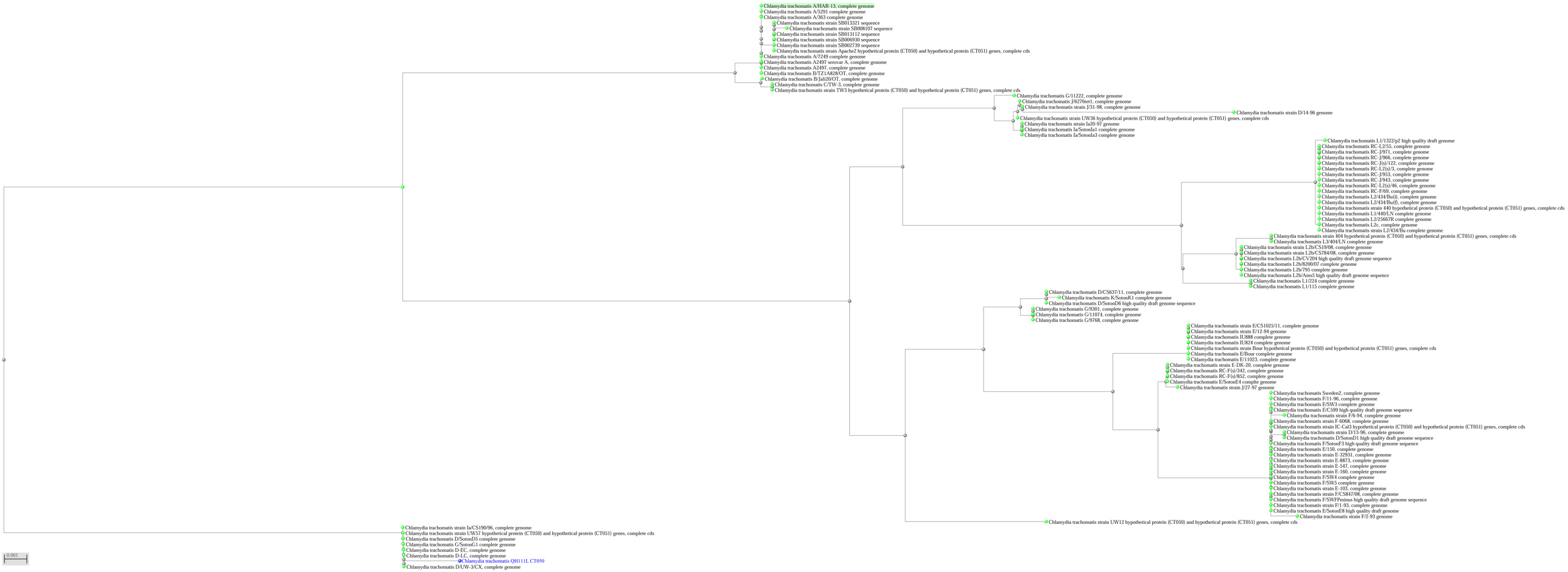

Supplement: Figure S4 — A fast-minimum-evolution tree produced by BLAST analysis of the QH111L CT050 gene. [file Image4.TIF]

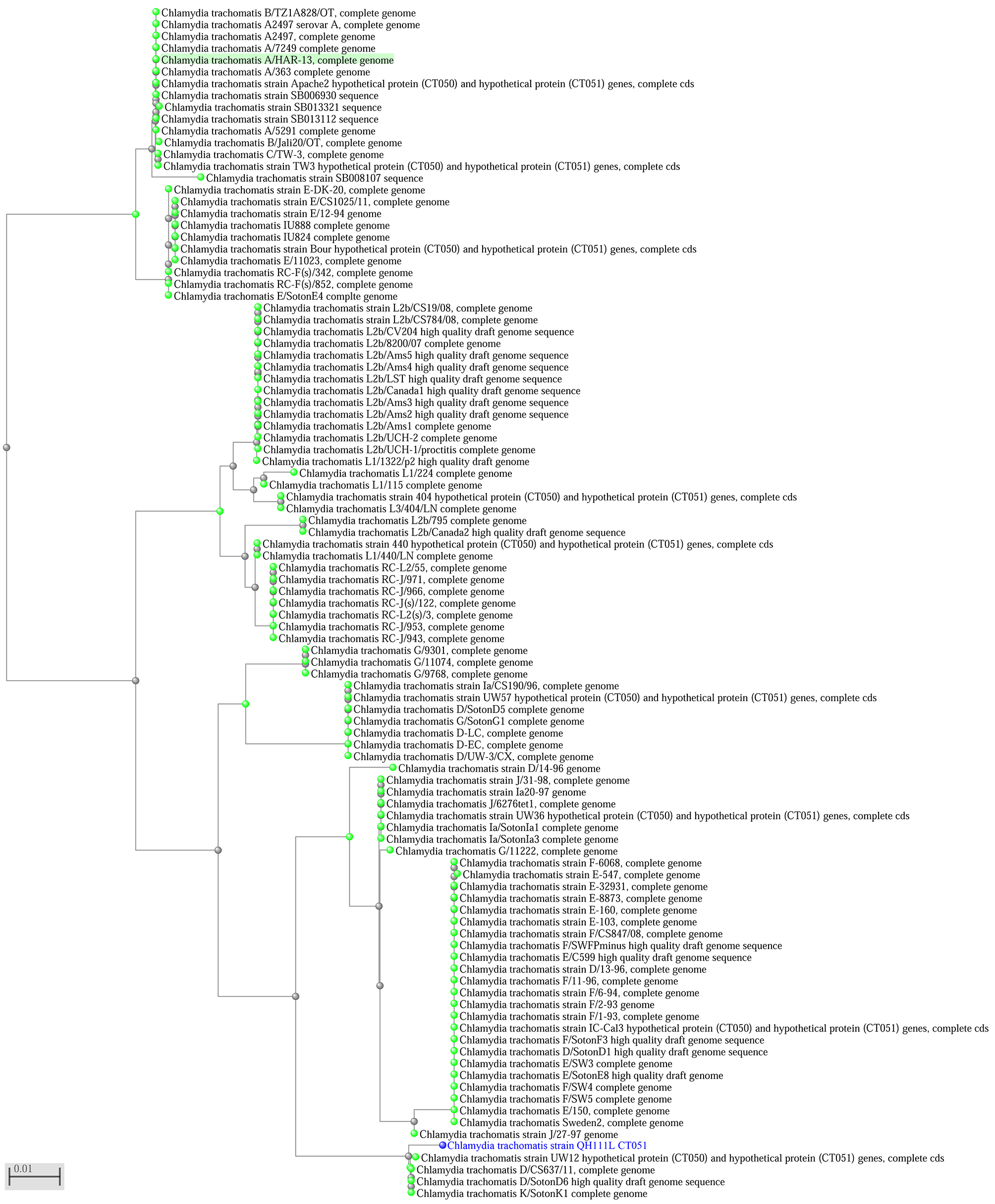

Supplement: Figure S5 — A fast-minimum-evolution tree produced by BLAST analysis of the QH111L CT051 gene. [file Image5.TIF]

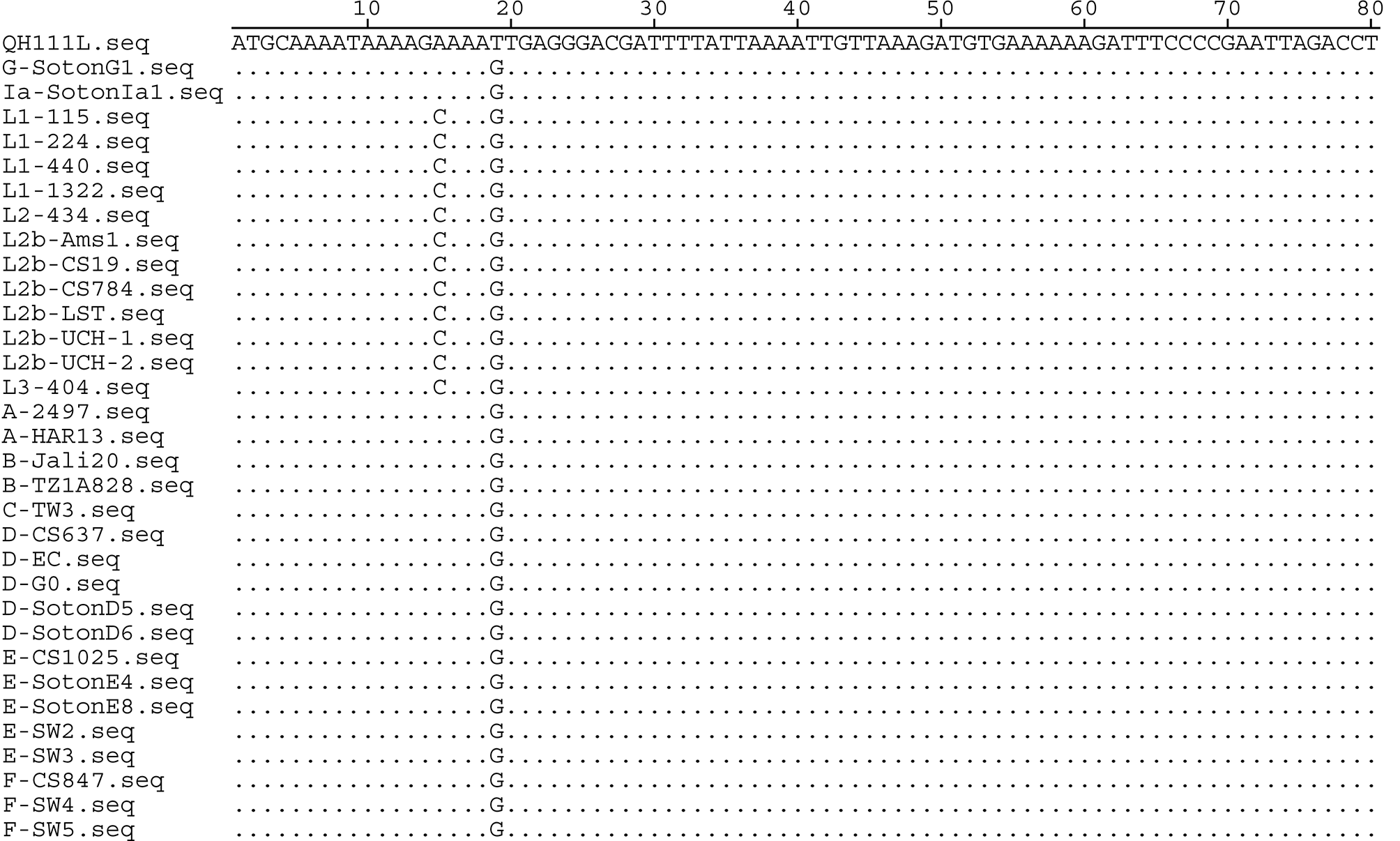

Supplement: Figure S6 — Partial alignment of available pgp4 sequences from GenBank. [file Image6.TIF]

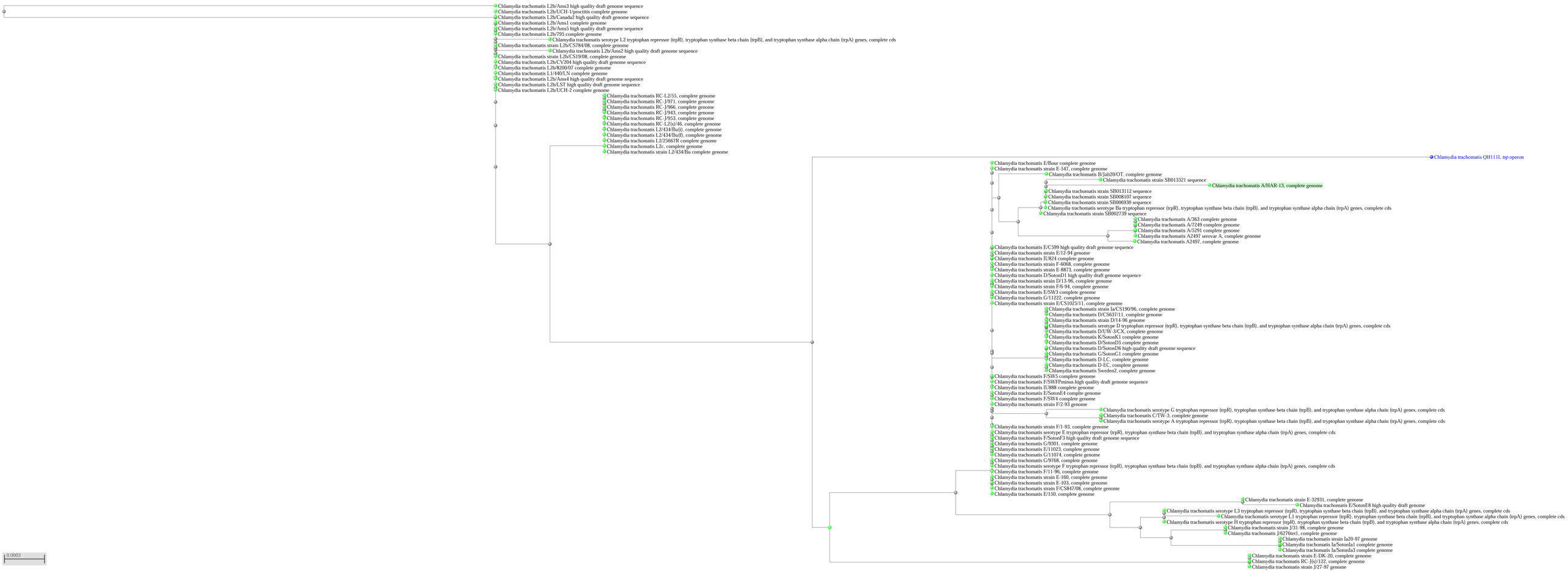

Supplement: Figure S7 — A fast-minimum-evolution phylogenetic tree produced by BLAST analysis of the QH111L trp operon. [file Image7.TIF]
